# Effect of Lignosulphonates on the Moisture Resistance of Phenol–Formaldehyde Resins for Exterior Plywood

**DOI:** 10.3390/ma17153715

**Published:** 2024-07-27

**Authors:** Sofia Gonçalves, Nádia T. Paiva, Jorge Martins, Fernão D. Magalhães, Luísa H. Carvalho

**Affiliations:** 1 LEPABE—Laboratory for Process Engineering, Environment, Biotechnology and Energy, Faculty of Engineering, University of Porto, Rua Dr. Roberto Frias, 4200-465 Porto, Portugal; up201808942@edu.fe.up.pt (S.G.); jmmartins@estgv.ipv.pt (J.M.); fdmagalh@fe.up.pt (F.D.M.); 2 ALiCE—Associate Laboratory in Chemical Engineering, Faculty of Engineering, University of Porto, Rua Dr. Roberto Frias, 4200-465 Porto, Portugal; 3 Sonae Arauco Portugal S.A., Lugar do Espido—Via Norte, 4470-177 Porto, Portugal; nadia.paiva@sonaearauco.com; 4 DEMad—Department of Wood Engineering, Instituto Politécnico de Viseu, Campus Politécnico de Repeses, 3504-510 Viseu, Portugal

**Keywords:** lignosulphonates, plywood, phenolic resins, methylolation

## Abstract

Phenol–formaldehyde (PF) resins remain the preferred adhesive for exterior plywood, as they confer these boards their extreme weather resistance. However, their high price and toxicity has made phenol alternatives, such as technical lignins, increasingly more attractive. While many works report the use of kraft lignin, the most commercially available form are lignosulphonates (LS). However, these lack industrial success and are associated with low moisture resistance. In the current study, lignosulphonate–phenol–formaldehyde (LPF) resoles were synthesized considering a phenol replacement of 30% (*w*/*w*). Two LS samples of softwood (SLS) and hardwood (HLS) origin were compared. These samples were previously methylolated to increase their reactivity. The effectiveness of the treatment was confirmed through the Automated Bonding Evaluation System. Plywood was manufactured and tested according to EN 314 class 3 for exterior conditions, which is seldom found in the literature. Although a 35% increase in shear strength is still necessary to comply with the standard, methylolated SLS was the most promising substitute, as it resulted in the highest board performance. Notably, when this sample was used without previous methylolation, the plywood boards suffered delamination during immersion in boiling water prior to shear testing. These results reinforce the need for the methylolation of LS to increase the weather resistance of plywood.

## 1. Introduction

The first veneer is suspected to have been manufactured at around 3000 B.C. in ancient Egypt. In fact, a type of plywood has been found in coffins dating back to this period, possibly using albumin glues. The modern form of plywood has been used since 1860 in a variety of different applications, initially in furniture items and later as a construction material. During World War I, plywood was used in the production of aircrafts [1,2]. Currently, plywood remains a highly valuable material. In fact, in 2021, the global plywood market revenue was at USD 52 billion [3].

**Figure 1:**
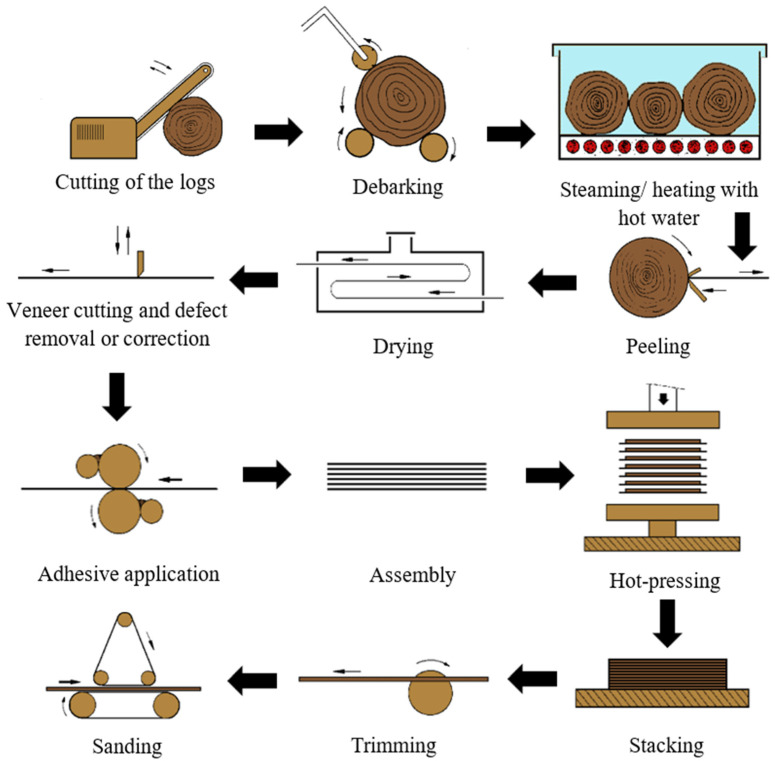
Plywood production process.

These wood-based panels consist of an assembly of layers glued together with the direction of the grain in adjacent layers usually at right angles [4,5]. The manufacturing process consists mainly of eight steps: log preparation, heating with steam/hot water, peeling, drying, veneer grading and cutting, adhesive application, assembly of veneer into panels, pressing, and finishing [6]. A schematic version of the plywood production process is shown in Figure 1.

The quality of the veneers largely influences the final plywood performance. Thus, the production process begins with the preparation of the logs. As the bark cannot be cut into useful veneer, it is removed. Then, the logs are heated with steam and/or hot water, increasing their flexibility and allowing them to easily transformed into veneers with less strains and internal stresses. The temperature of this process varies depending on the wood species [6].

Veneering follows as the logs are transformed into continuous sheets by rotating a bolt against a sharp knife. These sheets are then dried to a moisture content between 6 and 12%. Then, the veneer is graded, and the sheets are separated according to their quality. In this step, the patching of veneer sheets may also take place. Thus, defects, such as knots, are patched [6].

The adhesive application occurs on both sides of alternate veneers, usually through the means of a roller coater. The type of adhesive also depends on the final application of the boards, as phenol–formaldehyde resins are commonly used in exterior grade plywood, whilst urea-formaldehyde (UF) or melamine-urea-formaldehyde (MUF) resins are used in interior applications. The grammage of adhesive is selected according to the veneer wood species and the smoothness of its surface, the grade of the panel, as well as the applied adhesive [6]. For example, spread rates of 160 to 200 g/m^2^ can be applied; however, for low porosity veneers, rates of 130 to 140 g/m^2^ have been found to be appropriate. These adhesive formulations are composed of a resin, as well as fillers and/or diluents, which adjust wetting and avoid excessive adhesive penetration, allowing for a uniform joint thickness. For exterior grade plywood, CaCO_3_ or non-swelling fillers, such as coconut shell flour, are used in combination with phenol–formaldehyde resins. On the other hand, for interior applications, rye or wheat flour are used alongside UF or MUF resins. Nevertheless, an excessive amount of fillers may deteriorate the plywood’s performance [4,7].

In spite of the current advances in automation, plywood panels are mostly assembled manually [6]. Then, these are submitted to pressing, where pressures of 0.8 to 1.5 N/mm^2^ are commonly applied for softwood veneers; on the other hand, for hardwood species, these values can range from 1.5 to 2.5 N/mm^2^. Pressure requirements also vary according to the number of veneers and their thickness. Pressing temperatures have also been reported to range from 100 to 165 °C, depending on the chosen adhesive. These conditions result in adhesive temperatures at the bond line from 60 to 120 °C [4,8]. Lastly, the plywood is trimmed to size and sanded according to a target thickness [4]. A wide range of finishes may also be applied, such as non-slip surfaces and various colours [6].

As mentioned previously, plywood can be used for both interior, humid, and exterior applications [4]. Nevertheless, for exterior wood products, phenolic resins, usually resoles, are mostly applied. These resins allow these boards to retain their properties even after being repeatedly exposed to moisture, drying, or extreme weather [4,7].

In the synthesis of phenol–formaldehyde (PF) resins, phenols condense with formaldehyde at the 2-, 4-, or 6-position in the presence of acid or alkali catalysts. Thus, methylolphenol or phenolic alcohol is formed, and then dimethylolphenol is formed. The reaction proceeds in a second stage where methylol groups react with other available phenols or methylolphenols, resulting first in the formation of linear polymers and then in highly branched structures [9]. As numerous condensation products can be formed, the structure of these resins is still not fully understood [10].

The chemistry of phenolic resins is influenced by a series of factors such as the formaldehyde-to-phenol molar ratio, the used catalyst (acid, base, metal salt, or enzyme), the physical state of the resin (liquid, solid, dispersion), as well as its type (thermoplastic, named novolacs, or thermosetting and resols) [4].

**Figure 2:**
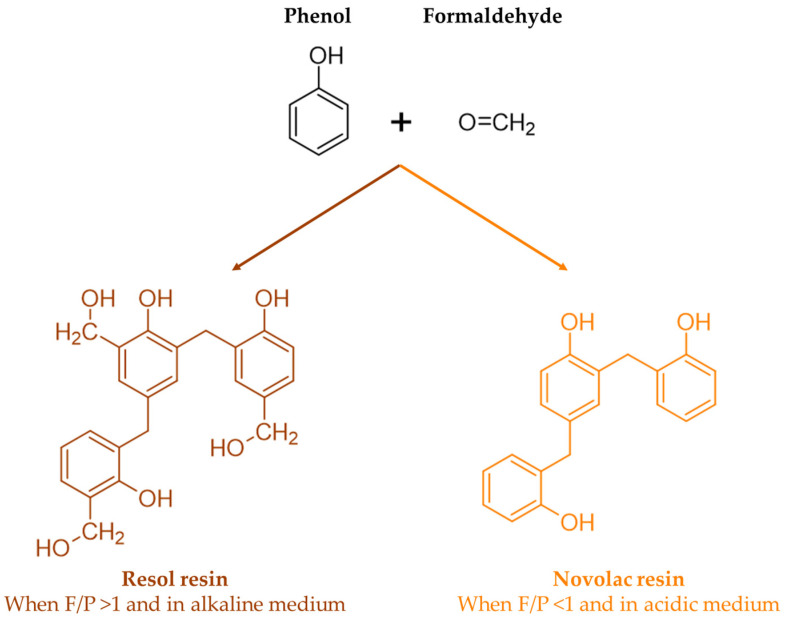
Structure of resol and novolac resins (adapted from [7]).

When a base catalyst combined with an excess of formaldehyde-to-phenol ratio is used, a resol resin is obtained. On the other hand, when less than an equimolar amount of formaldehyde is used under acidic conditions, a novolac resin is produced. These reactions are shown in Figure 2 [7].

Unlike resols, novolac resins contain no reactive methylol groups. Therefore, these resins need to be mixed with compounds that can release formaldehyde and form methylene bridges, such as paraformaldehyde or hexamethylenetetramine, in order to crosslink. On the other hand, acidification or heating causes resols to crosslink through their uncondensed phenolic alcohol groups and, possibly, through the release of formaldehyde by the breakdown of ether links [11].

The main advantages of PF resins are their resistance to water, weather, and high temperatures, as well as their high mechanical performance. Nonetheless, their high price and toxicity has made phenol alternatives, such as technical lignins, increasingly more attractive [9].

As the most abundant renewable source of phenolic compounds of natural origin, lignin has been increasingly researched as an eco-friendly alternative to petroleum-based compounds [12]. The large amounts of lignin waste have been proposed for adhesive production since the beginning of the wood pulping industry. However, only 1 to 2% of the global lignin production is used for obtaining value-added products. Lignosulphonates (LS), derived from the sulphite pulping process, account for 70% of the total market for commercial lignin [13,14,15,16]. Currently, the most successful industrial attempts comprise using lignin in combination with phenol–formaldehyde resins or urea–formaldehyde (UF) resins [17,18].

Many approaches have been suggested to increase the reactivity of lignin molecules and, consequently, facilitate their incorporation in resins. These include phenolation, hydrolysis, oxidation, treatment with ionic liquids, and, lastly, methylolation [19,20,21,22,23,24]. Lignin methylolation, or hydroxymethylation, introduces hydroxymethyl groups (–CH_2_OH) onto lignin molecules. This reaction usually takes place with formaldehyde in an alkaline medium. The resultant lignin can be directly incorporated as a phenol substitute in the synthesis of PF resol resins for wood adhesives [21].

Ghorbani and co-workers studied four commercial spruce LS for different sulphite pulping processes as partial 40% (*w*/*w*) phenol in lignin–phenol–formaldehyde (LPF) resol resins. Although displaying the lowest average MW and dispersity, sodium LS, when incorporated into the LPF resin for gluing beech veneer strips, resulted in the best curing and tensile shear strength development under hot pressing. On the other hand, calcium and magnesium LS were less suited as phenol replacements, since the obtained LPF adhesives had poor performance. The authors also claimed that the phenolation of sodium and ammonium LS, which had the most promising characteristics, did not significantly improve the performance of the obtained LPF resins [25]. Nevertheless, the moisture resistance of the LPF resins was not assessed, although this property is frequently reported to be compromised when LS are used in wood adhesives. These resins were also not applied in the production of plywood.

Studies have also shown that impurities in crude lignin, specifically elemental sugars, lowered the reactivity of lignin towards formaldehyde for the incorporation in LPF resins, thus leading to longer pressing times. The strength and water resistance of the obtained resins were also hindered [26,27].

Alonso and co-workers used methylolated softwood ammonium LS as a phenol substitute in the synthesis of a LPF resin. The optimum operating conditions for this synthesis were studied and defined as a phenol substitution of 35% (*w*/*w*), a NaOH-to-phenol and LS molar ratio of 0.6, and a formaldehyde-to-phenol and LS molar ratio of 2.5. The authors claimed that the obtained LPF resin complied with the specifications necessary for use in plywood. Its characteristics were also similar to those of the commercial PF resol resin used as a reference [28]. Nonetheless, this study did not include the production of plywood nor any testing of plywood samples.

Lastly, Akhtar et al. used herbaceous lignosulphonates as phenol substitutes in PF resins for exterior plywood. These boards where submitted to three separate pre-immersion treatments prior to shear testing: 24 h in water at room temperature, 2 h in water at 70 °C, and immersion in boiling water for 8 h. The authors concluded that the pretreatments did not significantly affect the shear testing results. However, the increase in LS content resulted in a significant decrease in board performance. For a 70% phenol substitution, a 50% decrease in shear load was observed. Nonetheless, the authors concluded that these boards obeyed Pakistani standard PS.871:1970 (plywood for general purposes) [29]. It should be noted that these boards were not assessed according to the more demanding current European standard EN 314-1 [30].

Other types of technical lignins have also been used as phenol substitutes in the production of plywood with higher degrees of success.

Ghorbani et al. achieved a 40% (*w*/*w*) phenol substitution using pine kraft lignin in exterior grade plywood, which complied with the requirements of EN 314-2 bonding class 3 for exterior conditions. When this lignin sample was previously methylolated, no significant improvements in terms of bonding strength were detected [31,32].

These studies all report up to 40% (*w*/*w*) phenol substitution without the loss of the resin’s performance. However, Kalami and co-workers have reported 100% (*w*/*w*) phenol substitution. In this study, the applied lignin was a byproduct of the bioethanol process obtained through a dilute acid pretreatment and the enzymatic hydrolysis of corn stover. When the LPF resin was used to produce poplar veneer single-lap-joint samples, there was no significant difference between the shear strengths made with the LPF adhesives and those made with the commercial phenol–resorcinol–formaldehyde resin. These conclusions were also maintained after a pretreatment in boiling water for 4 h, subsequent drying at 65 °C for 20 h, and a final immersion in boiling water for another 4 h [33]. Although these single-lap-joint samples were submitted to a treatment similar to EN 314-2 bonding class 3 for exterior conditions, plywood was not manufactured.

Patents have also been filed by companies, such as UPM, which report a phenol substitution of up to 50% (*w*/*w*) in plywood production. For this purpose, kraft lignin was previously modified through alkylation, phenolation, and methylolation [34]. UPM also claims that their lignin products can replace up to 80% (*w*/*w*) of phenol in PF resins for plywood whilst meeting the requirements of EN 314-2 bonding class 3 for exterior conditions. For this purpose, a high pressing factor of upwards of 240 s/mm was employed, whereas values of about 35 to 145 s/mm have been reported in industrial plywood production [2,29,35].

In this study, lignosulphonate–phenol–formaldehyde resoles were synthesized considering a phenol replacement of 30% (*w*/*w*) by LS. Two LS samples of softwood and hardwood origin were studied and compared. Their performance was evaluated, in terms of single-lap-joint bonding, through the Automated Bonding Evaluation System (ABES). Then, the most promising were used to produce plywood samples, whose performance was assessed according to EN 314-2 bonding class 3 for exterior conditions. This study aims to address a gap in the literature where kraft lignin is more commonly studied as a phenol substitute, while LS are dismissed for having poor moisture resistance. Similarly noteworthy, plywood was manufactured and tested according to the current European standard EN 314, which is seldom taken into consideration in the literature. As such, this study effectively addresses the implications of using LS as a partial phenol replacement in exterior-grade plywood.

## 2. Materials and Methods

### 2.1. Materials

Thick spent sulfite liquor (HLS) from the acidic magnesium-based sulfite pulping process of *Eucalyptus globulus* (hardwood) was supplied by Caima-Indústria de Celulose SA (Constância, Portugal). Spray-dried sodium lignosulphonate (SLS) (STARLIG^®^NA98S) from the sodium bisulfite pulping process of *Picea abies* (softwood) was supplied by LignoStar International BV (The Hague, The Netherlands). Poplar veneers were supplied by Laminar—Indústria de Madeiras e Derivados, S.A. (Vila Nova de Gaia, Portugal). Unless stated otherwise, all other chemicals were provided by Euroresinas—Indústrias Químicas, S.A. (Sines, Portugal), a company from Sonae Arauco Group.

### 2.2. Physico-Chemical Characterization of the LS Samples

For the determination of the dry matter content, the samples were dried in a ventilated oven at 105 °C until a constant mass was reached [36]. The ash content of the samples was determined gravimetrically after ignition in a muffle furnace at 525 °C, according to ISO 1762 [37].

For the spent sulphite liquor sample, the pH, density, and viscosity were also determined. Further, pH values were evaluated using an InLab Routine Pro combined pH glass electrode with an integrated temperature probe (Mettler Toledo, Columbus, OH, USA). The density and viscosity determinations were conducted using a hydrometer (Ludwig Schneider, Wertheim, Germany) and a Brookfield Model DV-II + viscometer with spindle 2 at 60 rpm (AMETEK Brookfield, Middleboro, MA, USA), respectively.

In order to determine the lignosulphonate content, 0.5 g of HLS was diluted with deionized water in a 1000 mL volumetric flask. In this case, the pH was previously adjusted to 5. A similar solution was prepared for the SLS, taking into account the solids content of the HLS. Then, 0.5 mL of these solutions was transferred to a quartz cuvette and diluted to 3.0 mL with deionized water [38]. The LS content of the samples was calculated from the absorbance at a wavelength of 232.5 nm, considering an extinction coefficient of 24.5 L g^−1^ cm^−1^ [39,40]. A GENESYS 10S UV-Vis spectrophotometer (Thermo Scientific, Waltham, MA, USA) was used.

### 2.3. Fourier Transform Infrared Spectroscopy (FTIR)

The infrared spectra were recorded using a VERTEX 70 FTIR spectrometer (Bruker, Billerica, MA, USA) in the transmittance mode with a high-sensitivity DLaTGS detector at room temperature. The samples were measured in ATR mode, with an A225/Q PLATINUM ATR Diamond crystal (Bruker, Billerica, MA, USA) with a single reflection accessory. The spectra were recorded from 4000 to 500 cm^−1^ with a resolution of 4 cm^−1^. The samples were previously dried at 105 °C.

### 2.4. Resin Synthesis

The resins were manufactured in round bottom flasks of 2000 mL equipped with a thermometer and mechanical stirrer. Temperature control was accomplished by means of a heating blanket. The synthesis process was essentially divided into two stages: methylolation and condensation.

The synthesis process began with the addition of phenol, water, and an appropriate amount of 50% (*w*/*w*) NaOH resulting in a pH of 9 to 10. Then, methylolation began with the addition of formaldehyde 50% (*w*/*w*) over a 30 min time period whilst maintaining the temperature at 60 °C. A formaldehyde-to-phenol ratio between 2.5 and 3.5 was considered. The mixture was then heated slowly to 95 °C and left to react for 15 min. Thereafter, the condensation began at 85 °C with the addition of 50% (*w*/*w*) NaOH, resulting in a pH of 10.5 to 12.

At this stage, the condensation of the resin was allowed to progress until a viscosity of about 250 to 450 cP was reached. Lastly, the reaction was stopped through cooling of the reaction mixture to room temperature.

When LS were used as a partial phenol substitute, they were added alongside phenol, considering a 10, 20, or 30% (*w*/*w*) substitution rate. This was determined considering that the concentration of LS in the HLS and SLS sample was 33% and 80%, respectively. The formaldehyde content of the resins was also reduced and determined according to a formaldehyde-to-LS solids ratio between 0.15 and 0.20.

### 2.5. Methylolation

Firstly, the jacketed reactor was loaded with the LS sample, and deionized water was added to adjust the final concentration of lignosulphonate in the reaction medium to approximately 280 g/L. Then, the pH was adjusted to 9.6 using a NaOH solution at 50% (*w*/*w*). Lastly, formaldehyde 50% (*w*/*w*) was added according to a formaldehyde-to-LS solids ratio of 0.17 and the reaction mixture was heated to the desired temperature. The mixture was left to react for 3 h at 60 °C or 1 h at 70 °C. 

### 2.6. Resin Characterization

- pH—The pH of the obtained resins was measured at 25 °C using a pH glass electrode with an integrated temperature probe.
- Viscosity (cP)—The viscosity of the resins was measured at a constant temperature of 25 °C using a Brookfield DV-II programmable viscometer.
- Density (g·cm^−3^)—The determination of the resin’s density is based on the weight/volume ratio and was conducted using a hydrometer.
- Solids content (%)—The solids content was determined by drying 2 g of resin for 2 h in an oven at 135 °C. Three replicates were caried out for each resin.
- Free formaldehyde content (%)—The free formaldehyde content of the obtained resins was not determined. This was due to the rapid precipitation of the resins upon the addition of acid, which is required for the adjustment of a sample’s pH as stated in ISO 11402:2004 [41].
- Water tolerance (%)—For the determination of the water tolerance of the resins, 5 mL of resin was placed inside a test tube and water was added gradually. When the solution turned turbid, the consumed water volume was registered and the water tolerance was determined as follows:
  $$Water\;tolerance=\frac{V_{H_{2}O}}{5}\times 100$$
  where $V_{H_{2}O}$ is the volume, in mL, of water added.
- Shelf-life (days)—The increase in viscosity of the resins over the course of several days at 25 °C was measured using a Brookfield DV-II programmable viscometer. The shelf-life of the resins was established as the number of days required for a viscosity above 700 cP to be reached.
- Automated Bonding Evaluation System (ABES)—The bonding performance to a wood substrate of the manufactured resins was evaluated by testing the samples in shear mode. For this test, two wood veneer strips (*Fagus sylvatica*), 0.5 mm thick, 20 mm wide, and 117 mm in length, were used. These strips were previously stored at a controlled temperature (25 °C) and relative humidity (65%) in order to stabilize them and achieve an equilibrium moisture content between 8 and 11% (dry basis). These wood test pieces were glued manually together with a spatula (10 mg in 100 mm^2^) with the resin formulation (composed of the resin, wheat flour, and calcium carbonate). After the set temperature was reached, the strips were mounted in the system with an overlapping area of 20 × 5 mm^2^ and pressed together at 1.2 N·mm^−2^ for a chosen period of time. The system is digitally controlled and pneumatically driven, and a standard loading rate of 1 kN·s^−1^ was used [42].

### 2.7. Plywood Production

Plywood samples were manufactured using poplar wood veneers bonded with the LPF resins. The adhesive mixture was composed of resin, wheat flour, and calcium carbonate. Five-layer plywood with a size of 50 cm × 50 cm were assembled by spreading the glue mixture on the veneer surface with a glue spread of 180 g/m^2^. The adhesive mixture was spread on top of the first sheet and on the bottom of the third, fourth, and fifth veneer. The plywood was pressed in a parallel plate hot-press, ITALPRESSE GL/90 (ITALPRESS, Bergamo, Italy), at 130 °C for 10 min, considering a target thickness of 10.5 mm. As a control, plywood bonded with a commercial PF adhesive was also prepared.

### 2.8. Plywood Characterization

**Figure 3:**
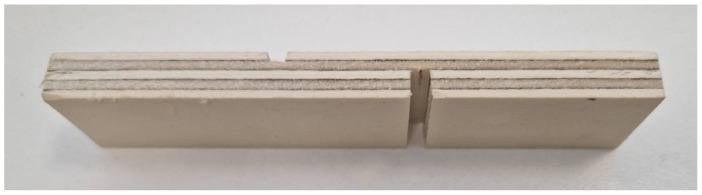
Plywood sample for shear testing according to EN 314.

The following physico-mechanical properties were studied in accordance with their respective European standards: density (EN 323), moisture content (EN 322), formaldehyde release through the gas analysis method (EN ISO 12460-3), modulus of elasticity and bending strength (EN 310), and bonding quality (EN 314-1). Before testing, the test pieces were subjected to the pretreatments according to class 3—exterior conditions (EN 314-2) [30,32,43,44,45,46]. Thus, the plywood samples, shown in Figure 3, were immersed in water for 24 h at 20 °C. Then, these where immersed in boiling water for 4 h and dried for 16 to 20 h at 60 °C. Afterwards, the samples were once again immersed in boiling water for 4 h. Lastly, the samples were cooled in water at 20 °C [32]. The requirements for EN 314-2 are shown in Table 1.

## 3. Results and Discussion

### 3.1. Characterization of LS Samples

Before the production of adhesives, the LS samples were characterized. These samples were named SLS and HLS due to their softwood and hardwood origin, respectively. The results of the physico-chemical characterization of these samples are shown in Table 2.

It was concluded that out of these two samples, SLS contains a higher content of lignosulphonates as well as a higher ash content. On the other hand, the results for the composition of HLS are consistent with those found in the literature [36,40,47,48,49].

**Figure 4:**
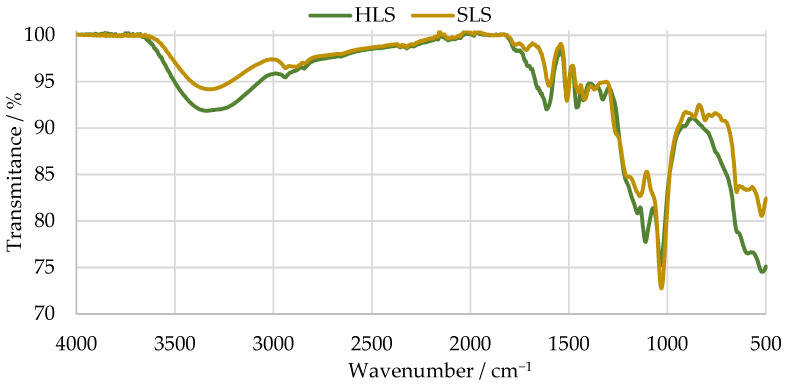
FTIR spectra of the SLS and HLS samples.

Then, the structure of theses samples was characterized by FTIR. The results are shown in Figure 4. The main bands were assigned according to [36,50,51] and are summarized in Table 3.

The spectra of both samples present bands characteristic of LS samples near 1034 cm^−1^. Peaks at around 1209 and 644 cm^−1^ indicate the presence of sulphonic groups [50,52]. Peaks associated with aromatic ring vibrations at around 1613, 1515, and 1426 cm^−1^ are also observed in both cases [50].

Nevertheless, only the SLS sample presents bands typical of G units at 1261, 1140, 865, and 809 cm^−1^. In contrast, the HLS sample presents bands characteristic of S units at 1328 and 1111 cm^−1^ [50]. The missing G bands of the HLS sample are explained by its high S:G ratio (81:19), which was previously reported in other studies [36,49]. On the other hand, the SLS sample is expected to be composed almost exclusively of G units [53]. This confirms the hardwood and softwood origins of the HLS and SLS samples, respectively.

Lastly, both spectra present a band of carbohydrate origin near 1710 cm^−1^. This is to be expected as both samples contain a considerable percentage of impurities, as shown in Table 2 by their low LS content [50].

Finally, it was concluded that, on a first analysis, SLS appears to be the most promising phenol substitute due to its higher purity and lower amount of substitution in the aromatic rings.

### 3.2. Shelf-Life and ABES Performance of Hardwood Lignosulphonates (HLS)

In an initial approach, 30% of total phenol weight present in the standard PF resin was replaced by the hardwood lignin sample consisting of spent sulphite liquor, resin 30% HLS PF. However, in order to increase the reactivity of LS, the samples were also previously modified by hydroxymethylation with formaldehyde. Two different operating conditions were used: lignin and formaldehyde were allowed to react at 60 °C for 3 h (resin identified as 30% 60mHLS PF) and at 70 °C for 1 h, (identified as 30% 70mHLS PF). These operating conditions were selected based on results from our previous work [54].

All of the manufactured resins were characterized regarding their pH, viscosity, solids content, and water tolerance. The results are displayed in Table 4. It was concluded that the incorporation of HLS increased the density of the resins. This increase is especially significant for the resins where the HLS were previously methylolated. It was also observed that the incorporation of LS significantly darkened the PF resin and increased its opacity. This was especially significant when the HLS sample was used. While the original PF resin was dark red, the LPF resins were nearly black.

**Figure 5:**
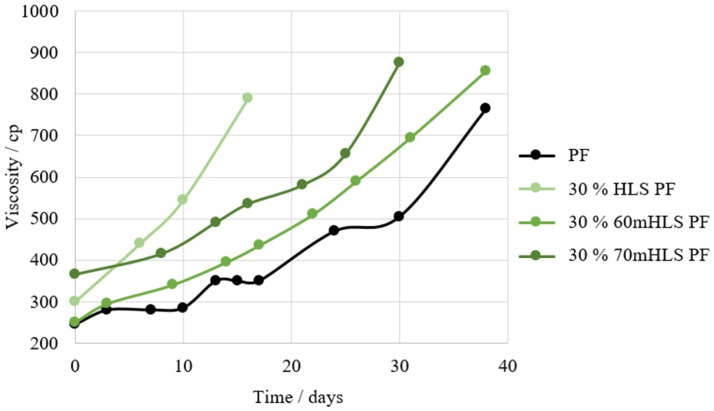
Shelf-life of the HLS PF resins in comparison to that of the commercial PF resin.

Then, the shelf-life of the resins was compared, using that of the commercial PF resin as reference. The results are shown in Figure 5.

It was concluded that the resins with the closest shelf-life to that of the standard PF resin are the resins where HLS was previously methylolated. Consequently, these are labelled as the most promising LPF resins. It is admitted that the previous methylolation of LS may have positively impacted the shelf-life of the resins through the reduction of their free formaldehyde content.

**Figure 6:**
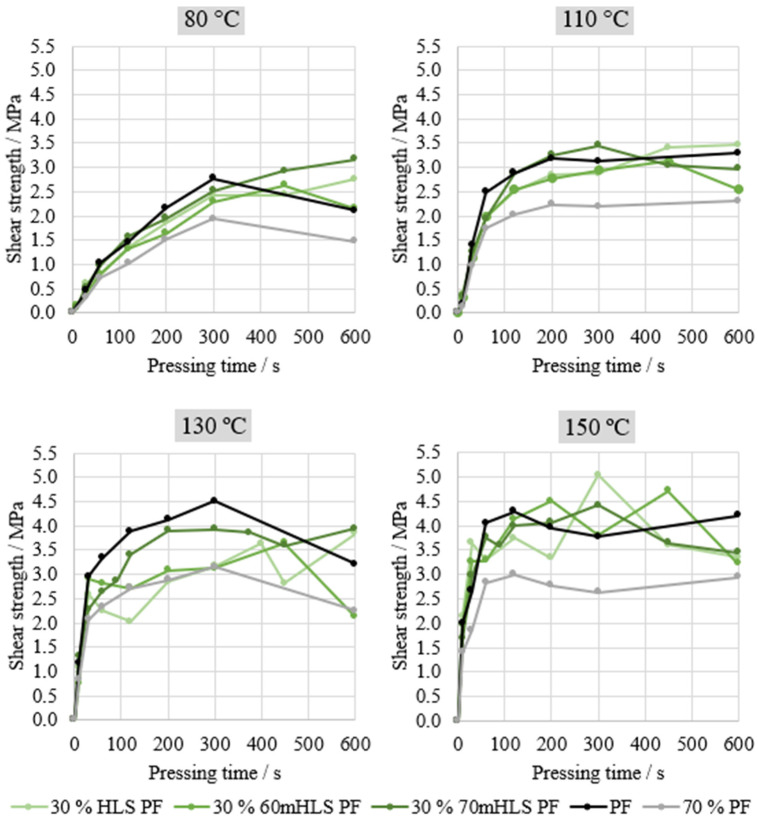
ABES results of the LPF resins manufactured with HLS.

Lastly, the performance of the manufactured resins was evaluated through ABES. Thus, the shear strength under tensile load of two veneer strips bound together by the manufactured resins was assessed. Four different pressing temperatures were assessed: 80, 110, 130, and 150 °C. This analysis was carried in order to select the most promising resins for plywood production, using the results of the commercial PF resin as reference, as shown in Figure 6. An additional curve is also shown—70% PF—simply computed to represent 70% of the commercial PF resin’s performance, in order to assess whether HLS are acting merely as an inert filler or actively contributing towards bonding performance.

As shown in Figure 6, the most significant differences in the performance of the resins are observed at 130 °C. In fact, it may be concluded that the unmethylolated HLS are acting merely as an inert filler. This can be concluded from the similarity between the curve corresponding to 70% of the PF resin’s performance and the curve of 30% HLS PF. When the sample was previously methylolated for 3 h at 60 °C, there appeared to be a slight increase in performance. However, this increase is only considered to be significant for the resin where LS were methylolated at 70 °C for 1 h. It should be noted that Ghorbani and co-workers also applied the automated bonding evaluation system to evaluate the effect of the methylolation of kraft lignin but did not observe any significant increases in the cure speed of the LPF resin nor any improvements in resin performance [31].

Consequently, the resin 30% 70mHLS PF was selected for plywood production, and the respective operating conditions for the methylolation of LS were also chosen for future trials.

### 3.3. Shelf-Life and ABES Performance of Softwood Lignosulphonates (SLS)

**Figure 7:**
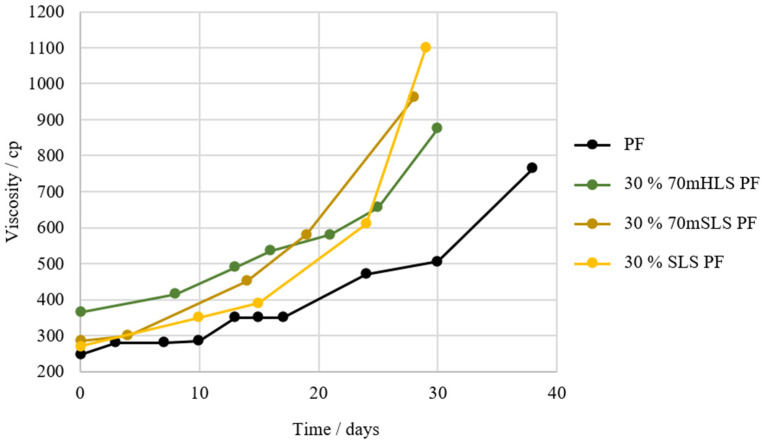
Shelf-life of resins 30% 70mSLS PF and 30% SLS PF in comparison to the commercial PF and 30% 70mHLS PF.

In another approach, the powder softwood lignosulphonate sample, SLS, was also tested as a phenol substitute so as to select the most promising lignin. Consequently, two different resins were manufactured once again, considering a 30% (*w*/*w*) phenol substitution: 30% 70mSLS PF and 30% SLS PF. In these resins, SLS were used with and without a previous methylolation, respectively. The results of the characterization of these resins are shown in Table 5 and their respective shelf-lives are shown Figure 7.

Once again, it is verified that the incorporation of LS in the PF resin results in an increase in viscosity. However, as shown in Figure 7, it appears that resins manufactured with SLS instead of HLS suffer a significantly faster increase in viscosity over time. This indicates that the synthesis of this resin still needs further optimization, namely through the reduction of the lignin-to-formaldehyde ratio, as SLS consumes less formaldehyde [54].

**Figure 8:**
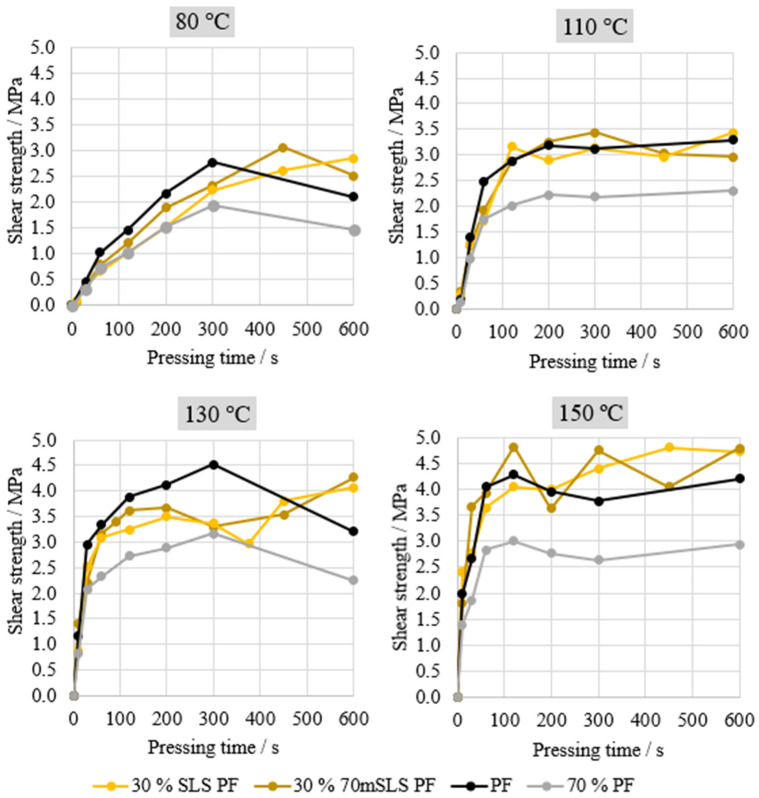
ABES results for resins manufactured with SLS.

Lastly, the performance of the resins synthesized with SLS was analysed and compared to the performance of the commercial PF resin. The results are shown in Figure 8.

Overall, both resins, 30% 70mSLS PF and 30% SLS PF, display performance similar to that of the commercial PF. However, at 80 °C a significant improvement was observed when SLS were previously methylolated at 70 °C for 1 h. Nevertheless, this behaviour is not verified at the remaining pressing temperatures. Consequently, both resins were selected for plywood production in order to conclude whether the selected lignin methylolation would result in a significant increase in board performance.

### 3.4. Plywood Bonding Performance—HLS vs. SLS

As mentioned previously, resins 30% 70mSLS PF and 30% SLS PF, alongside resin 30% 70mHLS PF, were selected as the most promising for plywood production. Control groups were also produced using the commercial PF resin. Plywood boards were manufactured with five layers, considering a pressing temperature of 130 °C and a pressing time of 10 min. Lastly, a commercial plywood board was also analysed as a control.

**Figure 9:**
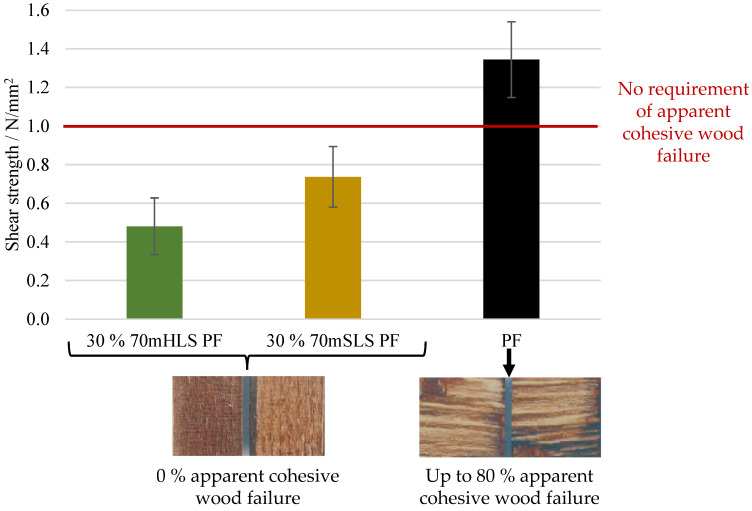
Results of the bonding quality of the LPF boards as well as the control lab PF board.

Firstly, the bond quality of the manufactured boards was analysed according to EN 314-2 for class 3—exterior conditions, and the appropriate pre-treatments were carried out. The results are shown in Figure 9.

As shown in Figure 9, the lab-scale PF boards display a shear strength value above 1 N/mm^2^ and, therefore, comply with the requirements of EN 314-2 regardless of the verified apparent cohesive wood failure. Despite fulfilling these requirements, it should also be noted that the lab-scale PF boards display a significantly lower (ANOVA *p*-value < 0.05) shear strength than that of an industrial board manufactured with the same resin—2.73 ± 0.97 N/mm^2^. These results highlight the limitations of the lab-scale process.

**Figure 10:**
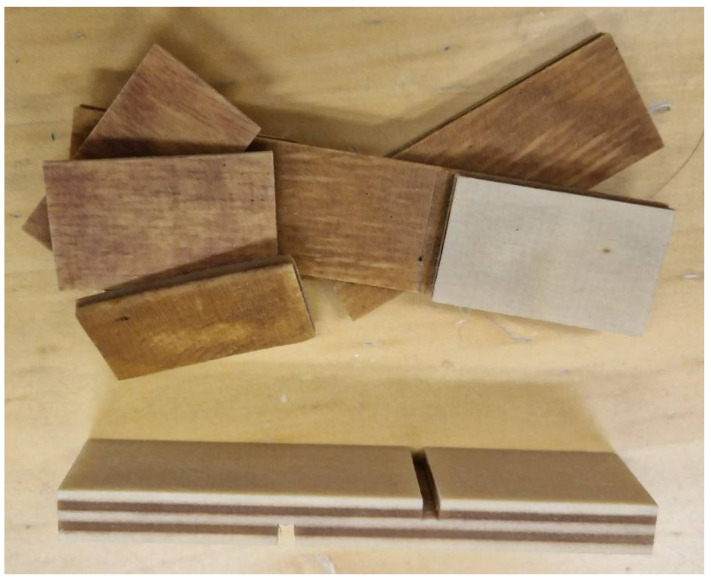
Delamination of the board samples manufactured using resin 30% SLS PF.

On the other hand, all lignin board samples display an average apparent cohesive wood failure of 0%. Consequently, the results of the LPF boards do not comply with the 1 N/mm^2^ requirement of EN 314-2 for this respective cohesive wood failure percentage. In contrast, the boards manufactured using resin 30% SLS PF suffered delamination during immersion in boiling water prior to shear testing, as shown in Figure 10. Therefore, it was concluded that the methylolation of the SLS sample significantly improved board performance. Lastly, SLS were also selected as the most promising phenol substitute as it led to a better board performance, although still below the requirements of EN 314-2. The lower performance of the more impure LS sample, HLS, is consistent with previous studies where impurities in crude lignin samples resulted in a decrease in water resistance and strength [26,27].

It should be noted that these results are inconsistent with those of Ghorbani et al. for other lignin samples, as these authors were able to comply with the requirements for EN 314-2 for class 3—exterior conditions at 40% (*w*/*w*) phenol substitution using kraft lignin. As previously mentioned, these authors also reported no significant improvements in performance when lignin was previously methylolated [31]. On the other hand, Akhtar and co-workers also reported a similar decrease in board performance when LS was used as a phenol substitute. However, unlike in our study, the authors claimed that the resin was water resistant [29].

**Figure 11:**
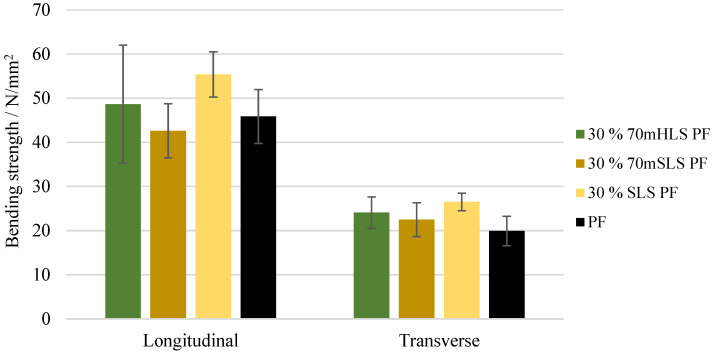
Bending strength for the boards manufactured with the LPF resins as well as the commercial PF resin.

**Figure 12:**
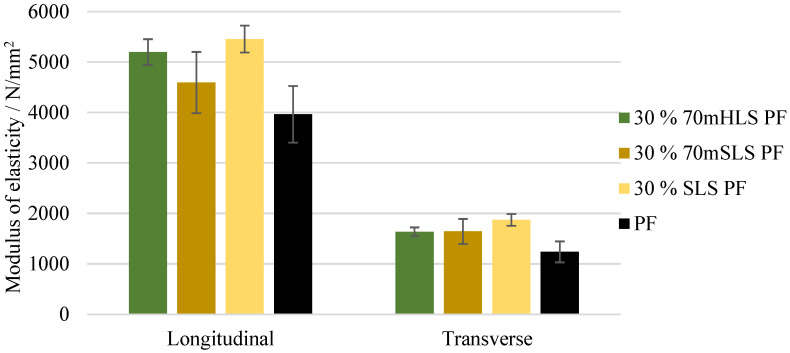
Modulus of elasticity for the boards manufactured with the LPF resins as well as the commercial PF resin.

**Figure 13:**
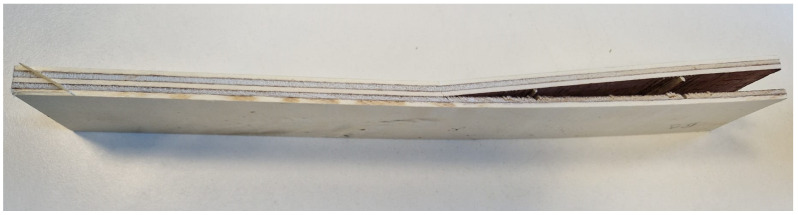
Board delamination within the glue lines as verified in the samples of the boards manufactured with the resins 30% 70mHLS PF and 30% SLS PF.

The bending strength and modulus of elasticity of these boards were also assessed. These results are depicted in Figure 11 and Figure 12, respectively. It was concluded that all boards display a similar performance to those of the commercial PF resin regarding their bending strength in the longitudinal direction (p>0.05). These results are consistent with those of Ghorbani et al. where kraft lignin was used as a phenol substitute [31]. However, a significant increase (p<0.05) in the modulus of elasticity was observed in the case of the boards manufactured with the resins 30% 70mHLS PF and 30% SLS PF. It should also be noted that in these cases, the board breakage occurred within the glue lines, as shown in Figure 13, instead of in the middle of the board.

**Figure 14:**
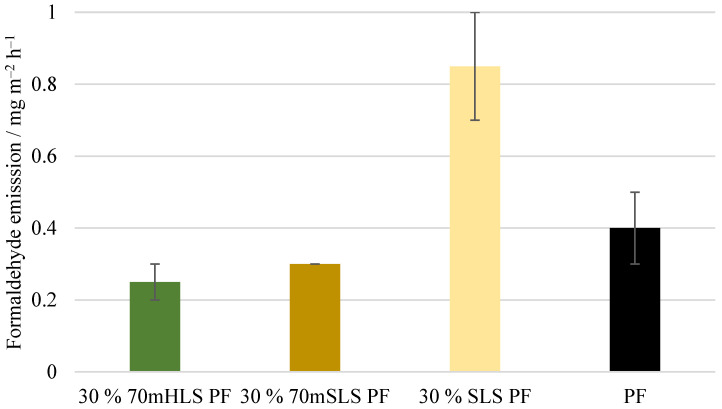
Formaldehyde emissions of the manufactured plywood boards.

Lastly, the formaldehyde emissions of the plywood boards, shown in Figure 14, were also analysed. These emission values are consistent with the value reported by UPM of 0.230 mg m^−2^ h^−1^ for 12 mm boards for LPF resins made using kraft lignin as a phenol substitute [35]. No significant differences (p>0.05) were detected between the emissions of the commercial PF boards and those of the methylolated lignin boards. However, for the unmethylolated SLS boards, a significant increase (p<0.05) in formaldehyde emissions was verified. Consequently, it was concluded that the previous methylolation of SLS was essential to promote the reaction between SLS and formaldehyde, allowing the reduction of formaldehyde emissions.

### 3.5. Plywood Bonding Performance—Effect of Phenol Substitution Amount

As SLS were selected as the most promising phenol substitute, the effect of using different substitution percentages on the performance of the LPF resin was studied. Consequently, two additional resins were manufactured using 10 and 20% of methylolated SLS. The results of the pH, viscosity, solids content, and water tolerance of these resins are displayed in Table 6. Visually it was observed that the increase in SLS content resulted in a progressively more opaque and darker resin.

**Figure 15:**
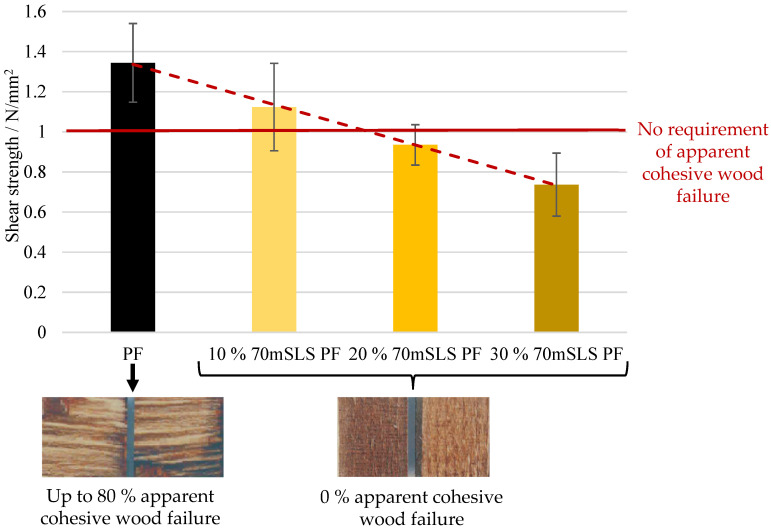
Results for the bond quality class 3 for the boards manufactured with the LPF resins with 10, 20, and 30% of methylolated SLS as well as the commercial PF resin.

These resins were then used to produce plywood whose performance was assessed as previously. The results of the bonding quality of the boards according to EN 314-2, class 3—exterior conditions, are shown in Figure 15. 

As shown in Figure 15, shear strength decreases linearly with the increase in methylolated SLS content. In fact, at 10% (*w*/*w*) phenol substitution, the shear strength of some board samples already fails to meet the 1 MPa specification (apparent cohesive wood failure of 0%). Therefore, it was concluded that the SLS sample significantly decreased (p<0.05) the moisture resistance of PF resins.

**Figure 16:**
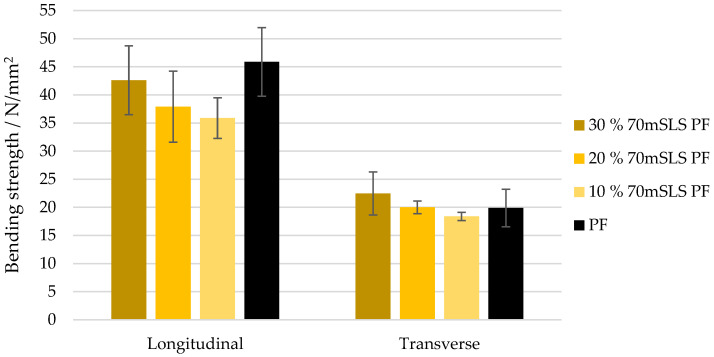
Bending strength for the boards manufactured with the LPF resins with 10, 20, and 30% of methylolated SLS as well as the commercial PF resin.

**Figure 17:**
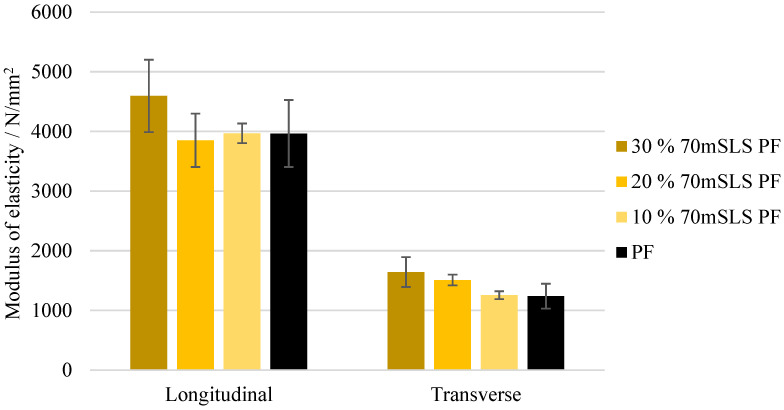
Modulus of elasticity for the boards manufactured with the LPF resins with 10, 20, and 30% of methylolated SLS as well as the commercial PF resin.

The bending strength and modulus of elasticity of these boards are shown in Figure 16 and Figure 17, respectively. As concluded previously, the LPF boards display a similar performance to those manufactured with the commercial PF resin. There appeared to be a slight increase in both the modulus of rupture and elasticity as the SLS content increases. However, this was only considered to be significantly different for the modulus of elasticity in the transverse direction (p<0.05).

**Figure 18:**
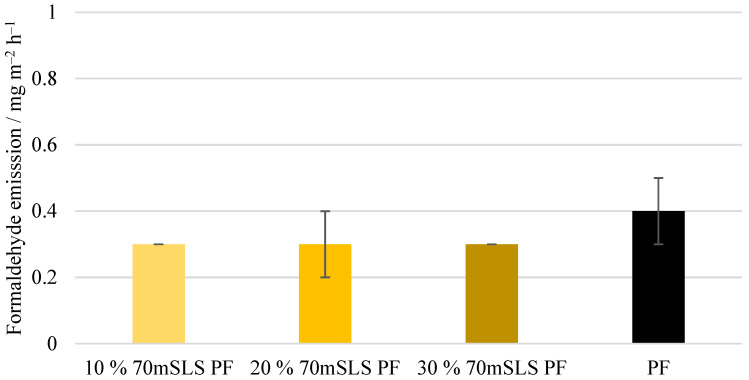
Formaldehyde emissions of the boards manufactured with the LPF resins with 10, 20, and 30% of methylolated SLS as well as the commercial PF resin.

Lastly, the formaldehyde emission (gas analysis value) of these boards was determined. As shown in Figure 18, no significant differences (p>0.05) were observed between the emissions of the SLS boards and that of the boards manufactured with the commercial PF resin.

To conclude, despite the fact that SLS appears to be a promising phenol substitute, further improvements still need to be made so that LPF boards comply with the requirements of EN 314-2, class 3—exterior conditions. Consequently, future studies should focus on optimizing the resin synthesis, namely through optimization of formaldehyde content. Nevertheless, this study also reinforces the need for lignin modification, prior to resin synthesis, in order to promote its chemical incorporation in the resin’s network. Future studies may also attempt other modifications to further increase lignin’s reactivity, such as phenolation and alkylation. Additional crosslinkers for lignin, such as furfural, may also be added so as to allow the increase of phenol substitution without the loss of the resin’s moisture resistance. In fact, furfural has been reported to increase the moisture resistance of LPF resins when added alongside formaldehyde in the resin’s synthesis. This improvement was justified by a more adequate penetration of the adhesive into the wood, as observed by a scanning electron microscope [55]. Consequently, in future studies, this analysis should also be carried out, as well as the optimization of the amount of fillers in the adhesive mixture.

## 4. Conclusions

Hardwood and softwood lignosulphonates (LS), HLS and SLS, respectively, were applied as partial phenol substitutes to produce lignosulphonate–phenol–formaldehyde resins for exterior plywood. At a 30% (*w*/*w*) phenol substitution, the physico-chemical characteristics of the original commercial phenol–formaldehyde resin were maintained.

Based on ABES (Automated Bonding Evaluation System) evaluation, three resins were selected as the most promising: the LPF resins using SLS as a phenol substitute without previous methylolation and the resins using SLS and HLS, previously methylolated at 70 °C.

At 30% (*w*/*w*) phenol replacement, the methylolated SLS sample resulted in the lowest decrease in plywood performance. Nonetheless, when this sample was used without prior methylolation, the plywood samples suffered delamination during immersion in boiling water prior to shear testing, as required in EN 314-2 bonding quality class 3 for exterior conditions.

None of the LPF boards obeyed the requirements of this standard. In fact, even at 10% (*w*/*w*) phenol substitution, a significant decrease in board performance was already observed.

Despite the insufficient performance of the plywood samples produced, this study provides new insights on the application of lignosulphonates in PF resins for the manufacture of exterior plywood. The results also reinforce the necessity of the modification of LS prior to its incorporation in phenolic resins in order to overcome their tendency to confer low moisture resistance to the final adhesive.

Future studies should attempt to further improve these results, testing other LS modifications, such as phenolation. Additional crosslinkers may also be added in order to increase phenol substitution without the loss of the LPF resin’s performance.

## Figures and Tables

**Table 1:** Requirements for EN 314-2 [32].

Shear Strength f_v_ (N/mm^2^)	Average Apparent Cohesive Wood Failure (%)
0.2 ≤ f_v_ < 0.4	≥80
0.4 ≤ f_v_ < 0.6	≥60
0.6 ≤ f_v_ < 1.0	≥40
1.0 ≤ f_v_	No requirements

**Table 2:** Physico-chemical characterization results of the HLS and SLS samples.

Parameter	SLS	HLS
Density/g/cm^3^	-	1.308
Viscosity/cP	-	32
pH	-	2.48
Dry matter/%	96.0	54.7
LS content/%	80.8	33.5
Carbohydrate content/%	<6 ^b^	12.6 ^c^
LS content/% ^a^	84.2	61.2
Ash content/% ^a^	22.6	12.2

^a^ on a dry matter basis; ^b^ in accordance with the supplier; ^c^ according to [47].

**Table 3:** FTIR absorption band assignments of the SLS and HLS samples [36,47,50,51,52].

Wavenumber (cm^−1^)	Band Origin and Comments	SLS	HLS
3420–3250	O-H stretch (phenolic and aliphatic OH)	3332	3332
3000–2842	C-H stretch in methyl and methylene groups	2938	2940
2850–2840	C-H stretching (OCH_3_)	2843	2844
2000–1650	Several bands from overtones and combinations (substituted benzene rings)	1769	1766
1738–1709	C=O stretch in unconjugated ketones, carbonyls, and in ester groups (frequently of carbohydrate origin)	1714	1704
1605–1593	Aromatic skeletal vibrations; C=O stretch	1601	1613
1515–1505	Aromatic skeletal vibrations	1510	1515
1470–1460	C-H deformations (asymmetric in CH_3_ and CH_2_)	1452	1461
1430–1422	Aromatic skeletal vibrations and C-H in-plane deformation	1418	1426
1370–1365	Aliphatic C-H stretch in CH_3_, not in OCH_3_; phenolic OH	1370	1367
1330–1325	S ring and G ring substituted in C_5_	-	1328
1270–1266	G ring; C=O stretch	1261	-
1260–1150	Sulphonic acids	1205	1209
1166	C=O in conjugated ester groups	-	1151
1140	Aromatic C-H in-plane deformation; typical for G units	1140	-
1128–1125	Aromatic C-H in-plane deformation (S units); secondary alcohols; C=O stretch	-	1111
1080–1010	Characteristic LS peak at 1037 cm^−1^; sulfonic acids; deformation of aromatic C-H and C-O in primary alcohols; C=O stretch unconjugated	1030	1034
858–853	C-H out-of-plane in positions 2, 5, and 6 of G units	865	-
832–817	C-H out-of-plane in positions 2, 5, and 6 of G units	809	-
700–600	Sulphonic acids	648	644

**Table 4:** Characteristics of the LPF resins manufactured with the HLS sample.

	PF	30% HLS PF	30% 60mHLS PF	30% 70mHLS PF
pH	12.4	11.9	12.1	12.4
Viscosity (cP)	245	300	250	365
Solids content (%)	42.16 ± 0.23	43.01 ± 0.07	44.57 ± 0.10	43.36 ± 0.12
Density (g/cm^3^)	1.192	1.209	1.221	1.222
Water tolerance (%)	>2000			

**Table 5:** Characteristics of resins 30% 70mSLS PF and 30% SLS PF in comparison to the commercial PF and 30% 70mHLS PF.

	PF	30% 70mHLS PF	30% SLS PF	30% 70mSLS PF
pH	12.4	12.4	12.1	12.1
Viscosity (cP)	245	365	270	285
Solids content (%)	42.16 ± 0.23	43.36 ± 0.12	44.46 ± 0.05	44.53 ± 0.05
Density (g/cm^3^)	1.192	1.222	1.217	1.218
Water tolerance (%)	>2000			

**Table 6:** Characteristics of resins the resins manufactured with 10, 20, and 30% of 70mSLS in comparison to the commercial PF.

	**PF**	**10% 70mSLS PF**	**20% 70mSLS PF**	**30% 70mSLS PF**
pH	12.4	12.2	12.3	12.1
Viscosity (cP)	245	265	285	285
Solids content (%)	42.16 ± 0.23	42.06 ± 0.09	42.32 ± 0.17	44.53 ± 0.05
Density (g/cm^3^)	1.192	1.192	1.201	1.218
Water tolerance (%)	>2000			

## Data Availability

The raw data supporting the conclusions of this article will be made available by the authors on request.
